# Carbon nanotube sponges filled sandwich panels with superior high-power continuous wave laser resistance

**DOI:** 10.1038/s41598-022-25829-4

**Published:** 2022-12-12

**Authors:** Wu Yuan, Kailu Xiao, Xianqian Wu, Jiangtao Wang, Te Ma, Hongwei Song, Chenguang Huang

**Affiliations:** 1 Key Laboratory for Mechanics in Fluid-Solid Coupling Systems, Institute of Mechanics, Chinese Academy of Sciences, Beijing, 100190 China; 2 School of Engineering Sciences, University of Chinese Academy of Sciences, Beijing, 100049 China; 3 Materials and Process Simulation Center, California Institute of Technology, Pasadena, CA 91125 USA

**Keywords:** Mechanical properties, Mechanical engineering

## Abstract

Effect of highly-porous and lightweight carbon nanotube sponges on the high-power continuous wave laser ablation resistance of the sandwich panel was investigated experimentally. As a comparison, thermal responses of monolithic plate, carbon nanotube film filled sandwich panel, unfilled sandwich panel and carbon nanotube sponge filled sandwich panel subjected to continuous wave laser irradiation were analyzed. Experimental results showed that the laser resistance of the carbon nanotube filled sandwich panel is obviously higher than the unfilled structure. The added failure time of the sandwich panel by filling the cores with the carbon nanotube sponge of unit mass was about 18 times and 33 times longer than that by filling with the conventional ablative and insulated material. It could be understood by the high thermal diffusion coefficient and latent heat of sublimation of the carbon nanotube sponge. During ablation by the continuous wave, the carbon nanotube sponge not only fast consumed the absorbed laser energy through phase change of a large-area material due to its high latent heat of sublimation, but also quickly dispersed the heat energy introduced by the continuous wave laser due to its high thermal diffusion coefficient, leading to the extraordinary laser ablation resistance.

## Introduction

Sandwich structures are widely used in engineering industries such as aerospace and transportation to realize the lightweight and multifunctional design^1–3^. In addition, it provides numerous open-cell cores for filling advanced materials to significantly improve its performance under different conditions^4–8^. Our previous study showed that filling lightweight ablative material into the void space of the core not only postponed the failure time but also decreased the extent of the damage of the continuous wave (CW) laser irradiated sandwich panels^9^. For the ablative material filled sandwich panel, high temperature phase change of carbon powder plays a leading role on laser resistance of the sandwich panel, and resin matrix mainly exert supporting effect on the carbon powder. Therefore, it may be a more efficient way to make the most of the pure carbon filled in the core on the dissipation of the heat energy to improve the laser resistance under the condition that structural weight is nearly not increased.

Carbon nanotube (CNT) structure is a kind of multifunctional nano-material with superior mechanical properties and electrical and thermal conductivity^10–17^. Currently a large quantity of CNT film and CNT sponges, which can be applied in engineering practice, can be fabricated. There is a considerable body of knowledge in the literature that addresses the properties of CNT sponges such as mechanical behavior, conductivity, and thermal insulation as well as its application in aspects of solar cell and phase-change material^11,17–23^. With advantages of bearing large deformation and anti-cycle failure, CNT sponges can be filled in the load-bearing sandwich structure to realize multifunctional design such as load bearing and thermal insulation^21^. Even though CNT sponge has a very low macroscopic thermal conductivity, the heat energy could transfer along the CNT direction very rapidly. As a result, CNT sponges could disperse the heat energy induced by the CW laser irradiation and delay the failure time of the sandwich panel.

Laser interactions with solid materials have received increasing attention in various conditions, including laser welding^24,25^, laser drilling^26^, laser cutting^27^ and laser processing^28^, and laser induced damages^9,29^. For the highly-porous materials, Chen et al.^30^ studied the interaction between the pulsed ultra-violet laser and the CNT sponge, and discussed the plasmatic property caused by pulse laser in CNT sponge. When a high-power CW laser beam is applied on the porous materials, the primary mechanisms of the damage are evaporation and expulsion of the material from the laser spot. In reality, the back surface temperature reaching the melting point is our main concern.

To investigate the effect of ultralight CNT sponges, the response of the laser irradiated sandwich panel of CNT sponges filled sandwich panel was studied experimentally. As a comparison, continuous laser irradiation experiments of monolithic plate, CNT film filled sandwich panel and unfilled sandwich panel were also carried out under the same conditions. Thermal infrared imager (TIC) and high-speed camera (HSC) were used to obtain the temperature distribution and failure process of back free surface. It’s observed that because of absorption effect of laser energy by high-temperature phase change and its diffusion effect on panel temperature field, filing CNT sponge could delay the failure time of the sandwich panel dramatically.

## Filler materials

CNT film and CNT sponge materials used in the experiment were provided by Suzhou Institute of Nano-Tech and Nano Bionics. Thickness and size of CNT film were 100 nm and 40 mm × 40 mm, respectively. The size of CNT sponge was 40 mm × 40 mm and the thickness is 8 mm. The density and porosity of CNT sponge were 5–10 mg/cm^3^ and > 99%, respectively. Macroscopic thermal conductivity coefficient of CNT sponge is lower than 0.15 W/(m·K) due to the high porosity.

## Experiments

Four types of structures were considered: monotholic plate, CNT film filled sandwich panel, unfilled sandwich panel and CNT sponge filled sandwich panel. To ensure identical areal density, the thickness of the monotholic plate was 1.8 mm and those of both front and back panels in the sandwich panel were 0.9 mm. The added mass by the CNT film and CNT sponge could be neglected when compared to the weight of the panel. For the CNT film filled sandwich panel, the two layers of the panel were contact directly. For the CNT sponges filled sandwich panel, the distance between the two panels was 8 mm.

**Figure 1:**
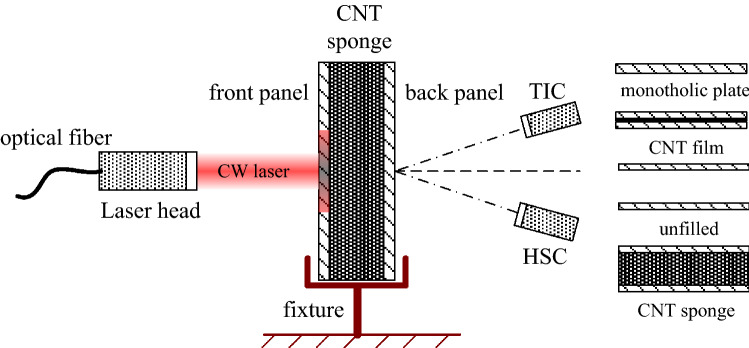
Schematic of the experimental setup and the types of specimens tested in the experiment.

Figure 1 shows the experimental setup. A TIC was placed at the back side of the sandwich panel to obtain the full-field temperature distribution. The resolution and the sampling frequency of the TIC were 420 × 640 pixel and 30 Hz, respectively. The temperature measurement range was 100–2700 °C. To get the dynamic damage evolution of the back surface, a HSC was also placed at the back surface of the specimen. The sampling frequency and resolution were 60 Hz and 1600 pixel × 1200 pixel, respectively. A IPG YLS 2000 W fiber laser operating at 1.07 μm was utilized as the laser source. In the laser irradiation experiment, 500 W output laser was adopted. By adjusting the distance from the specimen to the laser head, which is about 741 mm, the laser beam with a diameter of 5 mm was obtained.

## Results and discussions

### Temperature history and failure point

When used as a load-bearing structure of high-speed aircraft, the sandwich structure is usually faced with an external high-speed air flow or a substantial pressure difference between the front and back surfaces. Under this circumstance, the molten material is rapidly peeled off due to the mechanical erosion or the effect of internal pressure. Then, the laser is irradiated the components and parts inside the aircraft directly so as to result in serious damage. Therefore, the time for back surface of sandwich panel to reach melting point was defined in the experiment as the failure time.

**Figure 2:**
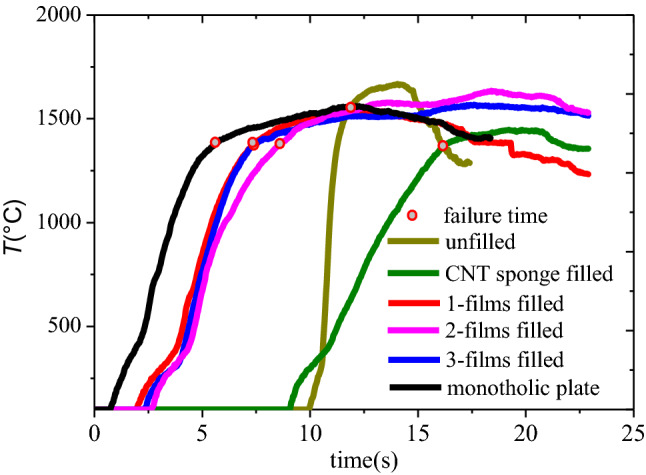
Comparison of temperature histories of monolithic plate, CNT film filled sandwich panel, unfilled sandwich panel and CNT sponge filled sandwich panel.

Figure 2 compares the laser spot center temperature histories of the back surfaces for different structures under CW laser irradiation. It can be found that due to high thermal conductivity along thickness direction, back surface temperature of the monolithic plate rose rapidly at the start of the irradiation time and reached melting point at about 5 s. Temperature on back surface maintained at melting-point temperature due to the surface tension.

For CNT film filled sandwich panel, obviously temperature rise didn’t happen on the back surface until 2.5 s because of thermal resistance effect between two panels and the absorbing effect of CNT film on the laser energy. As laser irradiation time increased, temperature rose rapidly and reached melting point at 7.3–8.5 s. In addition, temperature rising rate of CNT sponges filled sandwich panel after obviously temperature rise happened was identical with that of monolithic plate.

As stated in the reference^9^, heat energy generated by laser irradiation was mainly transmitted in the front panel before the front panel was penetrated by melting. Then, the back surface temperature rose rapidly after 10 s and reached the melting point at 11.9 s. Due to the small heat sink of the back panel, the temperature rise rate of unfilled sandwich panel was far greater than those of the monolithic plate and the CNT film filled sandwich panel. As a result, the structure is failed rapidly when the front panel is melted through.

For the CNT sponge filled sandwich panel, thermal response time was earlier than the unfilled sandwich panel due to conduction of the CNT sponge, but the temperature rising rate of the structure after obvious temperature rise happened was far lower than other three types. The time that the spot center temperature of the back surface reached the melting point was longer than that of the unfilled sandwich panel. Therefore, filling CNT sponges could significantly improve the laser resistance of the sandwich panel.

**Figure 3:**
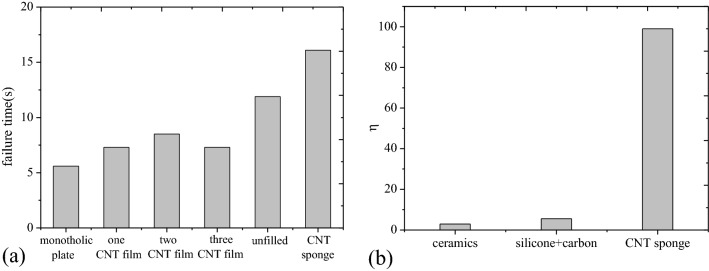
Effect of filler on the laser resistance of the sandwich panel. (**a**) Failure time, (**b**) ratio of increment of failure time to increment of structural weight due to addition of filler material. Laser irradiation behavior of the ceramic and the compound of silicone resin and carbon powder filled sandwich panel can be found in reference^9^.

Failure times of the four structures are shown in Fig. 3a. It is seen that splitting monolithic plate into two-layer thin panels and filling them with CNT films could improve the laser resistance of the structure. Increasing quantity of CNT film could not delay the failure time of the structure. Therefore, laser energy absorbed by CNT film could be neglected due to the ultra-thin thickness. The thermal resistance between the two panels resulted in the longer laser failure time. Enlarging the distance between the two panels could postpone thermal response time and failure time of the sandwich panel. However, after the front panel was melt through, temperature rising rate was too high, resulting in the rapid failure. Compared with the unfilled sandwich panel, the CNT sponge filled sandwich panel had short thermal response time. It had, however, higher laser resistance due to high latent heat of phase change of the CNT sponge. Here $\eta$ was defined as ratio of increment of failure time to increment of structural weight due to addition of filler material in unfilled sandwich panel:

1 $$ \eta = \frac{{\left( {t_{filled} - t_{unfilled} } \right)/t_{unfilled} }}{{\left( {m_{filled} - m_{unfilled} } \right)/m_{unfilled} }} $$

where $t_{filled}$ and $t_{unfilled}$ are laser failure time of filled and unfilled sandwich panels, respectively. $m_{filled}$ and $m_{unfilled}$ are weights of filled and unfilled sandwich panels, respectively. Figure 3b shows the influences of different fillers on CW laser failure strength of the sandwich panel. Laser irradiation experiment of the rest two materials can be found in reference^9^. It can be found the resisting efficiency of the CNT sponge is far superior to other two fillers. The added failure time by filling the CNT sponge of unit mass is about 18 and 33 times than that filling with the conventional ablative and insulated material.

### Full-field temperature and damage evolution

**Figure 4:**
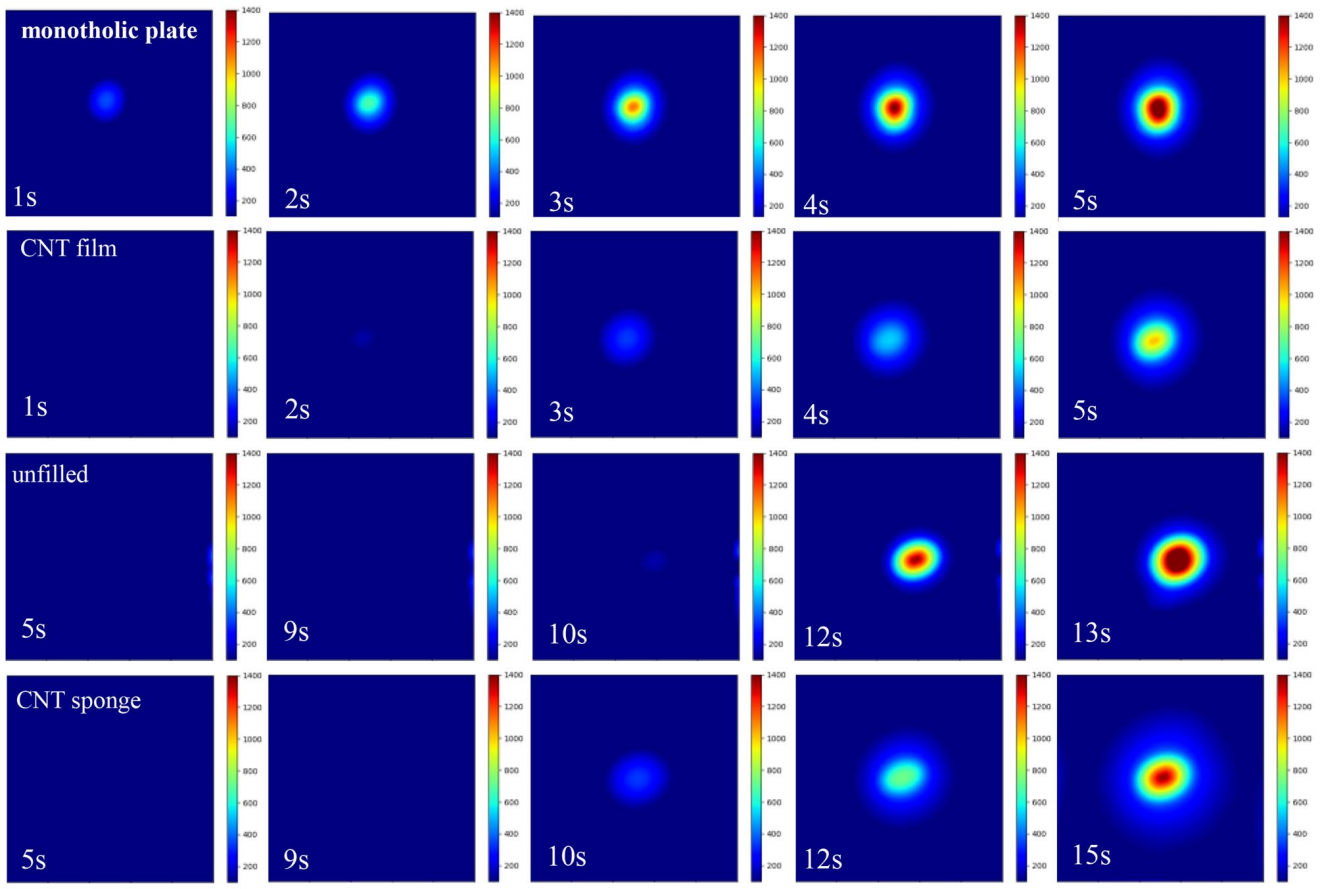
Temperature distribution in the back surface for monolithic plate, CNT film filled sandwich panel, unfilled sandwich panel and CNT sponge filled sandwich panel at different irradiation time, obtained by the thermal imaging camera.

Figure 4 give the dynamic evolution process of full-field temperature distribution for the four structures. Obvious temperature rise was happened at initial moment of laser irradiation on back surface of the monolithic plate. With the increase of the irradiation time, high-temperature region was continuously expanded and the highest temperature reached the melting point at about 5 s. For CNT film filled sandwich panel, the highest temperature on the back surface did not reach the melting point even at 5 s. No obvious temperature rise existed in the back surface of the unfilled sandwich panel even at 10 s due to the thermal resistance along the thickness direction. When compared to the unfilled sandwich panel, the CNT sponge filled sandwich panel had faster temperature response and low temperature rising rate, resulting in the later time for the temperature on back surface to reach melting point. The comparison of the full-field temperature distribution indicated that the high-temperature region on back surface of the CNT sponge filled sandwich panel was broader and the highest temperature was lower than that of the unfilled sandwich panel.

### Laser resistance mechanism of CNT sponge

**Figure 5:**
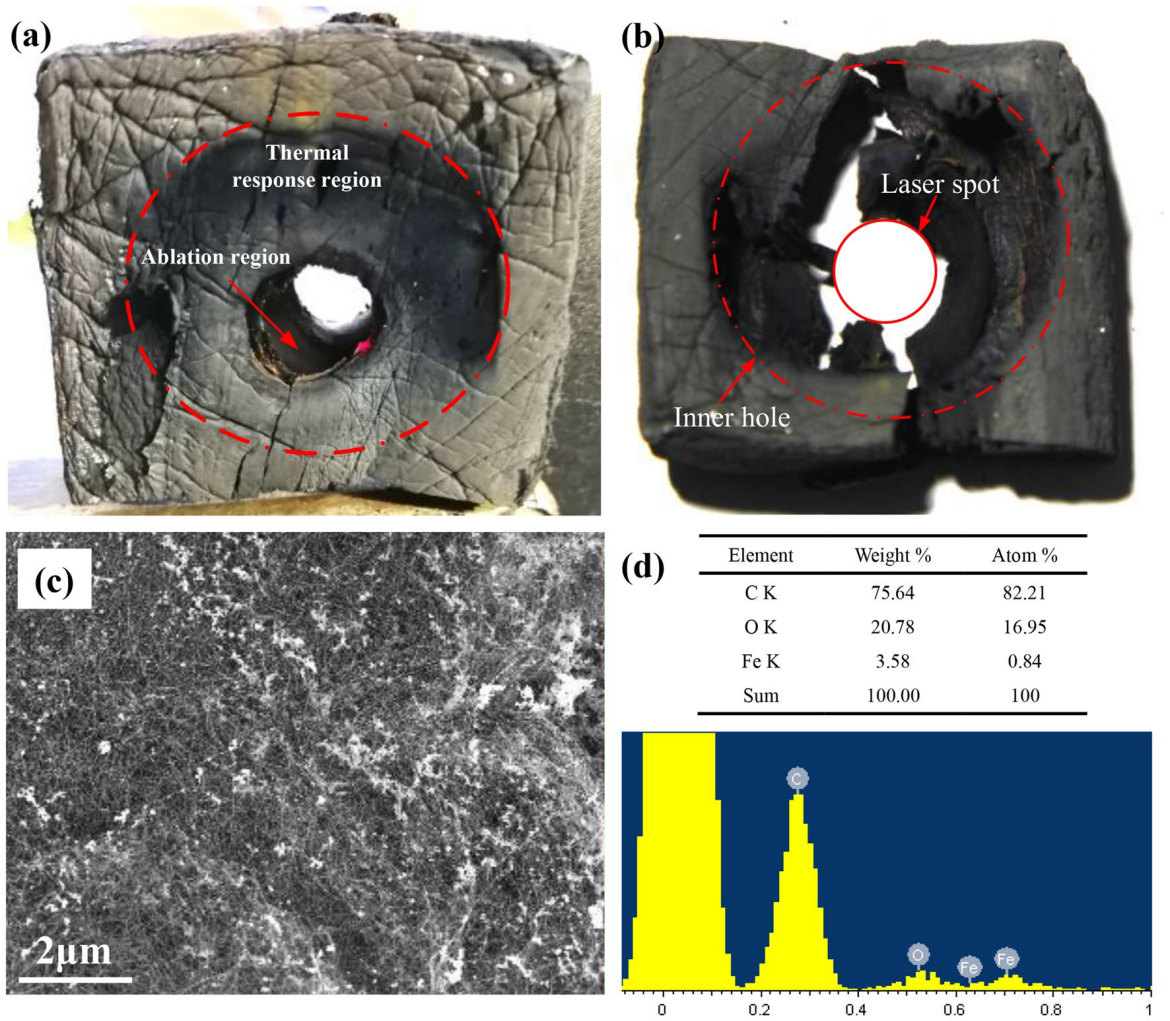
Ablation mechanisms of the CNT sponge: (**a**) outside ablation morphology, (**b**) inside ablation morphology, (**c**) SEM image of the contact region between the panel and the CNT sponge, (**d**) element content and EDS image.

Figure 5A shows the ablation morphology of the CNT sponge. The outer diameter of the ablation pit was approximate to laser spot. By peeling the external sponge region (black part) off the material, as shown in Fig. 5b, it could be found that the diameter of the inner ablation pit was about 3 times of the laser spot.

The heat diffusion coefficient is the rate that the temperature at one point in a body transmitted to another point, which can be expressed as:

2 $$ \alpha = \lambda /\rho c $$

Even though the thermal conductivity coefficient of the CNT sponge was low, due to its extremely low relative density, its heat diffusion coefficient was about 10 times of stainless steel material (5 × 10^–6^ m^2^/s). To sum up, strengthening effect of the CNT sponge on the laser resistance of the sandwich panel mainly includes two aspects: (1) latent heat of sublimation of carbon was far higher than other materials. Large quantity of the laser energy was absorbed by the CNT sponge through sublimation within diameter scope of laser spot; (2) CNT sponge dispersed the laser energy absorbed by the sandwich panel so that the temperature distribution on the panel was more uniform.

Figure 5c,d shows the SEM and EDS results for the CNT sponge that is directly contacted with the metallic panel. In the EDS results, attachment with shining colours on the CNT sponge was metallic solution. It indicates that the temperature on the contact surface between the CNT sponge and the metallic panel was higher than the melting temperature of the stainless steel. However, due to the large heat sink of the panel, the temperature of CNT sponge on the contact surface was lower than the sublimation temperature of the carbon.

## Conclusions

Experimental study on the laser resistance of CNT sponge filled sandwich panel was carried out. Dynamic evolution of temperature and ablation morphology on back surface of CW laser irradiated specimen was obtained using TIC and HSC. The ablation morphology and ablation products were observed by the SEM and EDS. Experimental results showed that the resisting efficiency of the CNT sponges to the sandwich panel under CW laser irradiation is dramatically superior than that of the traditional ablative and insulated materials. During the laser ablation process, in view of high thermal diffusion coefficient, on the one hand, phase change of large-area CNT sponge could absorb more laser energy. On the other hand, the CNT sponge dispersed structural temperature rise caused by laser irradiation so that the temperature distribution was more uniform, resulting in higher laser resistance of the sandwich panel. Due to the superior multifunctional performance, this kind of structures can be used in the thermal protection systems and load bearing structures.

## Data Availability

The datasets used and/or analysed during the current study available from the corresponding author on reasonable request.

## References

[1] Chai GB, Zhu S (2011). A review of low-velocity impact on sandwich structures. P. I. Mech. Eng. L-J Mat..

[2] Liu WL (2016). Design and ballistic penetration of the ceramic composite armor. Compos. Part B-Eng..

[3] Wang YP, Gong XL, Xuan SH (2018). Study of low-velocity impact response of sandwich panels with shear-thickening gel cores. Smart Mater. Struct..

[4] Han B (2015). Foam filling radically enhances transverse shear response of corrugated sandwich plates. Mater. Design.

[5] Ni CY (2015). Perforation resistance of corrugated metallic sandwich plates filled with reactive powder concrete: Experiment and simulation. Compos. Struct..

[6] Yan LL (2013). Compressive strength and energy absorption of sandwich panels with aluminum foam-filled corrugated cores. Compos. Sci. Technol..

[7] Yazici M, Wright J, Bertin D, Shukla A (2014). Experimental and numerical study of foam filled corrugated core steel sandwich structures subjected to blast loading. Compos. Struct..

[8] Yungwirth CJ, Radford DD, Aronson M, Wadley HNG (2008). Experiment assessment of the ballistic response of composite pyramidal lattice truss structures. Compos. Part B-Eng..

[9] Yuan W (2018). High-power laser resistance of filled sandwich panel with truss core: An experimental study. Compos. Struct..

[10] Baughman RH, Zakhidov AA, de Heer WA (2002). Carbon nanotubes—The route toward applications. Science.

[11] Erbay C (2015). Three-dimensional porous carbon nanotube sponges for high-performance anodes of microbial fuel cells. J. Power Sources.

[12] Liu JL (2014). High-performance flexible asymmetric supercapacitors based on a new graphene foam/carbon nanotube hybrid film. Energy Environ. Sci..

[13] Ma TY, Dai S, Jaroniec M, Qiao SZ (2014). Graphitic carbon nitride nanosheet-carbon nanotube three-dimensional porous composites as high-performance oxygen evolution electrocatalysts. Angew. Chem. Int. Ed..

[14] Mittal G, Dhand V, Rhee KY, Park SJ, Lee WR (2015). A review on carbon nanotubes and graphene as fillers in reinforced polymer nanocomposites. J. Ind. Eng. Chem..

[15] Wang DY (2015). Highly active and stable hybrid catalyst of cobalt-doped FeS_2_ nanosheets-carbon nanotubes for hydrogen evolution reaction. J. Am. Chem. Soc..

[16] Youn DH (2014). Highly active and stable hydrogen evolution electrocatalysts based on molybdenum compounds on carbon nanotube-graphene hybrid support. ACS Nano.

[17] Zhao WQ (2014). Elastic improvement of carbon nanotube sponges by depositing amorphous carbon coating. Carbon.

[18] Gui XC (2011). Recyclable carbon nanotube sponges for oil absorption. Acta Mater..

[19] Han DY, Mei H, Xiao SS, Cheng LF (2018). A direct chemical vapor infiltration route for a carbon nanotube/silicon carbide thermal protection system. J. Alloy Compd..

[20] Lin HT, Yang G, Tsao YYT, Liu YF, Yu C (2018). Ionic liquid treated carbon nanotube sponge as high areal capacity cathode for lithium sulfur batteries. J. Appl. Electrochem..

[21] Lin ZQ (2016). Carbon nanotube sponges, aerogels, and hierarchical composites: Synthesis, properties, and energy applications. Adv. Energy Mater..

[22] Zhang Q, Liu J (2018). Sebacic acid/CNT sponge phase change material with excellent thermal conductivity and photo-thermal performance. Sol. Energy Mater. Sol. C.

[23] Zhong J (2013). Carbon nanotube sponges as conductive networks for supercapacitor devices. Nano Energy.

[24] Froend M (2017). Fiber laser welding of dissimilar titanium (Ti-6A1-4V/cp-Ti) T-joints and their laser forming process for aircraft application. Opt. Laser Technol..

[25] Zhou L (2017). Influence of laser offset on laser welding-brazing of Al/brass dissimilar alloys. J. Alloy Compd..

[26] Marimuthu S (2017). An experimental study on quasi-CW fibre laser drilling of nickel superalloy. Opt. Laser Technol..

[27] Moradi M, Mehrabi O, Azdast T, Benyounis KY (2017). Enhancement of low power CO_2_ laser cutting process for injection molded polycarbonate. Opt. Laser Technol..

[28] Gan ZT, Yu G, He XL, Li SX (2017). Numerical simulation of thermal behavior and multicomponent mass transfer in direct laser deposition of Co-base alloy on steel. Int. J. Heat Mass Transf..

[29] Boley CD (2010). Interaction of a high-power laser beam with metal sheets. J. Appl. Phys..

[30] Chen JK (2015). Pulsed ultra-violet laser interactions with ultra-low-density porous carbon nanotube sponges. Carbon.

